# Clopidogrel, ticagrelor, prasugrel or an alternation of two P2Y12 in patients with acute myocardial infarction with cardiogenic shock

**DOI:** 10.3389/fcvm.2023.1266127

**Published:** 2024-01-03

**Authors:** Vojko Kanic, Gregor Kompara

**Affiliations:** Division of Internal Medicine, Department of Cardiology and Angiology, University Medical Center Maribor, Maribor, Slovenia

**Keywords:** clopidogrel, prasugrel, ticagrelor, P2Y12 combination, cardiogenic shock, myocardial infarction, outcome, bleeding

## Abstract

**Background:**

Data are lacking on the effects of the alternation of P2Y12 receptor antagonists (P2Y12) on bleeding and outcome in patients with myocardial infarction (MI) with cardiogenic shock (CS). We compared the effects of different P2Y12 and alternation of P2Y12 (combination) on bleeding and outcome in patients with MI and CS.

**Methods:**

Data from 247 patients divided into four groups: clopidogrel, ticagrelor, prasugrel, and the combination group, were analyzed. The association between P2Y12 and bleeding as well as 30-day and one-year mortality was examined.

**Results:**

The highest bleeding rate was observed in patients in the combination group, followed by the clopidogrel, ticagrelor, and prasugrel groups [12(50%) patients, 22(28.2%), 21(18.3%) and 4(13.3%), respectively; *p* = 0.003]. Bleeding occurred with a similar frequency in the combination and clopidogrel groups (*p* = 0.081), but more frequently than in the ticagrelor and prasugrel groups (*p* = 0.002 and *p* = 0.006, respectively). Bleeding rates were similar in patients receiving P2Y12 alone (*p* = 0.13). Compared to clopidogrel, both ticagrelor and prasugrel had a lower bleeding risk (aOR: 0.40; 95% CI: 0.18–0.92; *p* = 0.032 and aOR: 0.20; 95% CI: 0.05–0.85; *p* = 0.029, respectively) and the combination had a similar bleeding risk (aOR: 2.31; 95% CI: 0.71–7.48; *p* = 0.16). The ticagrelor and prasugrel groups had more than an 80% and 90% lower bleeding risk than the combination group (aOR: 0.17; 95% CI: 0.06–0.55; *p* = 0.003 and aOR: 0.09; 95% CI: 0.02–0.44; *p* = 0.003, respectively). The unadjusted 30-day and one-year mortality were highest in the clopidogrel group, followed by the ticagrelor, prasugrel, and combination groups (44(56.4%) and 55(70.5%) patients died in the clopidogrel group, 53(46.1%) and 56(48.7%) in the ticagrelor group, 12(40%) and 14(46.7%) patients died in the prasugrel, and 6(25%) and 9(37.5%) patients died in the combination group; *p* = 0.045 and *p* < 0.0001. After adjustment for confounders, the P2Y12 groups were not independently associated with either 30-day (*p* = 0.23) or one-year (*p* = 0.17) mortality risk.

**Conclusion:**

Our results suggest that the choice of P2Y12 was not associated with treatment outcome. The combination of P2Y12 increased bleeding risk compared with ticagrelor and prasugrel and was comparable to clopidogrel in patients with MI and CS. However, these higher bleeding rates did not result in worse treatment outcomes.

## Introduction

In patients with cardiogenic shock (CS) due to myocardial infarction (MI), percutaneous coronary intervention (PCI) is the standard recommended therapy (1). In addition, antithrombotic treatment is essential to prevent peri-PCI and later thrombotic events (2–8). In patients with CS, multiple challenges with antiplatelet action remain (9). The antiplatelet effect of orally administered drugs is delayed by gastroparesis, delayed intestinal absorption, and slower metabolism of the drugs due to compromised hemodynamics in cardiogenic shock, and after morphine administration (3, 5–7, 10). In addition, oral administration of the drugs may be problematic (9). The data on P2Y12 receptor antagonists (P2Y12) in patients with CS are sparse and still inconclusive (2, 3, 9). The combined results of two randomized trials showed that there is no difference between the P2Y12 (2) while pooled analysis of randomized and retrospective studies showed a better outcome with potent P2Y12 (3, 9). The data on bleeding are also controversial and inconclusive (9). Some have found no difference in bleeding (3, 9, 11, 12), whereas others have found a lower risk of bleeding with ticagrelor and a similar risk with prasugrel compared with clopidogrel (2). Randomized trials have numerous exclusion criteria and do not always capture the actual problems of individual patients. Some data show that almost 40% of patients with CS do not receive P2Y12 in everyday medical practice (6, 13) and even in randomized trials more than 30% do not receive P2Y12 (2). In daily practice, especially in patients with CS who are unable to communicate, patients sometimes receive a combination of P2Y12 receptor antagonists (not simultaneously). This usually occurs for two reasons-patients with STEMI received clopidogrel in pre-hospital settings (or were already on clopidogrel), which was then switched to ticagrelor or prasugrel during/after the procedure or later in hospital, or patients with MI received ticagrelor/prasugrel during initial treatment not knowing that they were previously receiving oral anticoagulant medications. Potent P2Y12 are usually later switched to clopidogrel in combination with oral anticoagulants. The combination of P2Y12 in the acute setting, when heparin/bivalirudin and GP IIb/IIIa receptor antagonists are also used, is expected to be associated with bleeding, a known predictor of worse outcome (1, 3). These patients have mostly been excluded from randomized trials or have not been evaluated separately, and there are no data on how the combination of oral P2Y12 receptor antagonists affects bleeding and outcome in patients with CS, in whom drug administration, absorption, metabolism, and efficacy differ from other patients with MI. The aim of our study was to evaluate the bleeding rate and 30-day and one-year mortality of different oral P2Y12 and their combination in patients with cardiogenic shock due to myocardial infarction.

## Materials and methods

The cohort of the present single-center retrospective observational study was recruited from patients with MI who underwent PCI between 2010 and 2018 (potent P2Y12 were not previously available) at the University Medical Center Maribor, Slovenia, a tertiary referral hospital with a 24/7 PCI service. Of 6,578 consecutively screened patients with MI who underwent PCI, we identified 381(5.8%) patients with CS. Patients who did not receive P2Y12 [134 (35.2%) patients] were excluded. The final patient cohort comprised 247 eligible patients. The patients were divided into four groups according to the P2Y12 received—clopidogrel [78(31.6%) patients], ticagrelor [115(46.6%) patients], prasugrel [30(12.1%) patients] and a group with modified P2Y12 therapy [24 (9.7%) patients] who received clopidogrel and prasugrel or clopidogrel and ticagrelor or ticagrelor and prasugrel. We did not subdivide patients in the latter group according to which P2Y12 was originally administered and which was later administered because there were too few patients in each group. These four groups were compared, and in-hospital bleeding and all-cause mortality were assessed at 30 days and one year.

Group P2Y12 data were provided for all patients, and data on all other essential patient and procedural characteristics were at least 94.7% complete. Ascertainment of end points was 100% complete. Data on the dates of death were provided by the Slovenian National Cause of Death Registry. The study was approved by the local institutional ethics committee (UKC-MB-KME-59/19), and all methods were performed in accordance with the requirements of the Declaration of Helsinki.

### Patients and definitions

The diagnosis of MI was made according to published guidelines, including a typical history of chest pain, diagnostic electrocardiographic changes, and serial elevations of cardiac biomarkers, and patients were treated according to published guidelines for the management of MI (1, 14, 15). Patients were eligible for analysis if they suffered MI with CS. The criteria for CS were a systolic blood pressure of ≤90 mm Hg for ≥30 min or the use of catecholamines to maintain a systolic blood pressure of >90 mm Hg, clinical signs of pulmonary congestion, and signs of end-organ hypoperfusion. Thrombolysis In Myocardial Infarction (TIMI) flow grades were used to assess coronary blood flow (16). Anemia was defined according to the World Health Organization recommendations: a serum hemoglobin level of <130 g/L in men and <120 g/L in women (17). Bleeding events were classified using the Bleeding Academic Research Consortium (BARC) definition and BARC 3,5 bleeding were used (18). For mechanical circulatory support, an intra-aortic balloon pump (IABP) was most commonly used. Extracorporeal membrane oxygenation (ECMO) was used in only two (1.4%) patients, both of whom died.

### Pharmacological treatment with P2y12 receptor antagonists

The use of P2Y12 was left to the discretion of the treating physician. Administration of more potent P2Y12 in addition to the clopidogrel loading dose given in the pre-hospital setting was not common but was left to the discretion of the operator or attending physician as was administration of clopidogrel instead of prasugrel/ticagrelor in patients who were on anticoagulation therapy or needed anticoagulation therapy.

### Study end points

The end points of the study were BARC 3, 5 in-hospital bleeding and all-cause 30-day and one-year mortality.

### Statistical methods

The patients were divided according to the P2Y12 received into four groups and these groups were compared. The Kolmogorov–Smirnov test was used to assess normal distribution. Differences between the groups in baseline clinical, angiographic, and procedural characteristics were compared with the two-sample *t*-test, Mann–Whitney test, or the Jonckheere–Terpstra test depending on whether the data followed the normal distribution for continuous variables and the chi-square test or Fischer's exact test for categorical variables, as appropriate. End-point events that occurred during the follow-up period were counted and their rates were compared among the groups. The cumulative incidence rates of the unadjusted one-year mortality were estimated by the Kaplan–Meier method and compared by the logrank test. Binary logistic regression models were performed using the Enter mode to determine the possible association between P2Y12 and bleeding and 30-day mortality, and Cox regression analysis was used to determine hazard ratios (HR) as estimates of one-year mortality. In addition to age and sex, the models for bleeding were adjusted for variables that had a significant univariant association (*p* < 0.05) with in-hospital bleeding [mechanical ventilation, resuscitation prior to PCI, glomerular filtration rate (GFR), anemia on admission, renal replacement therapy, and P2Y12 groups]. In addition, variables based on previous literature reports and experience that these factors are known to influence bleeding (radial access, GP IIb/IIIa receptor antagonists, bivalirudin, oral anticoagulant therapy) were added to the model. In addition to age, sex, and bleeding, the models for 30-day and one-year mortality were adjusted for variables with univariant association (*p* < 0.05) with 30-day mortality (mechanical ventilation, resuscitation prior to PCI, arterial hypertension, TIMI flow 0/1 after PCI, PCI of the right coronary artery, PCI of the circumflex artery, systolic blood pressure on admission, GFR, anemia on admission, and P2Y12 groups). Variables known to be associated with survival (diabetes and hyperlipidemia) were also included. The clopidogrel group was used as the reference group. ORs and HRs were calculated using a model stratified by P2Y12 groups. Only mechanical ventilation on admission was included as a variable in the calculations. All included variables had a variance inflation factor (VIF) < 1.8. Adjusted odds and hazard ratios (HR) for all four P2Y12 groups were calculated. Data were analyzed with SPSS 21.0 software for Windows (IBM Corp., Armonk, NY). All *p*-values were two-sided, and values less than 0.05 were considered statistically significant.

## Results

The oldest patients were those on clopidogrel (70.8 ± 1.7 years) or ticagrelor (67.3 ± 12.0 years), but the P2Y12 combination group (62.2 ± 10.3 years) and especially the prasugrel group (57.9 ± 10.9 years) were younger (*p* = 0.01). Patients taking clopidogrel were not only older but also more likely to have lower GFR and to be anemic on admission compared to the prasugrel and ticagrelor patients. Clopidogrel patients were resuscitated prior to PCI more frequently than prasugrel patients (*p* = 0.018) and tended to be resuscitated more frequently than ticagrelor patients (*p* = 0.056). They were also less likely to suffer a STEMI (*p* = 0.010), but more likely to have TIMI 0/1 flow after PCI than prasugrel patients (*p* = 0.034), and they tended to receive more oral anticoagulants than prasugrel patients (*p* = 0.059) and definitely more than ticagrelor patients (*p* = 0.021). The ticagrelor group was less likely to have had a previous myocardial infarction (*p* = 0.03) and less likely to receive oral anticoagulants (*p* = 0.03) than patients receiving a combination of P2Y12. Prasugrel patients were also less likely to receive oral anticoagulants than the P2Y12 combination group (*p* = 0.034). The P2Y12 combination group was more frequently treated with PCI LCX compared to the others (*p* = 0.033, *p* = 0.011, and *p* = 0.028 for the clopidogrel, ticagrelor, and prasugrel groups, respectively) and suffered more bleeding. Patient baseline and procedural characteristics, and outcome are shown in Table 1.

**Table 1:** Patient admission, procedural and outcome characteristics.

Variable	Clopidogrel	Ticagrelor	Prasugrel	P2Y12 combination	*p*
	*n* = 78 (31.6%)	*n* = 115 (46.6%)	*n* = 30 (12.3%)	*n* = 24 (9.7%)	
Age, years	70.8 (11.7)	67.3 (12.0)	57.9 (10.9)	62.2 (10.3)	<0.0001
Male sex	49 (62.8%)	76 (66.1%)	23 (76.7%)	17 (70.8%)	0.56
Diabetes mellitus	17 (21.8%)	27 (23.5%)	5 (16.7%)	6 (25.0%)	0.86
Hypertension	36 (46.2%)	45 (39.1%)	10 (33.3%)	7 (29.2%)	0.39
Hyperlipidemia	10 (12.8%)	21 (18.3%)	7 (23.3%)	6 (25.0%)	0.42
Chronic kidney disease	4 (5.1%)	5 (4.3%)	0 (0.0%)	0 (0.0%)	0.44
Previous MI	3 (3.8%)	4 (3.5%)	4 (13.3%)	4 (16.7%)	0.023
Previous stroke	4 (5.1%)	7 (6.1%)	0 (0.0%)	1 (4.2%)	0.58
Previous PCI/CABG	3 (3.8%)	6 (5.2%)	2 (6.7%)	1 (4.2%)	0.93
Oral AC therapy	10 (12.8%)	4 (3.5%)	0 (0.0%)	4 (16.7%)	0.009
Resuscitation before PCI	41 (52.6%)	44 (38.3%)	8 (26.7%)	9 (37.5%)	0.063
BMI, kg/m^2^	26.2 (24.1, 29.4)	27.7 (24.8, 31.9)	27.7 (25.7, 31.2)	26.6 (25.3, 28.7)	0.28
Hemoglobin, g/L	123.0 (108.7, 136.5)	135.0 (119.0,143.0)	134.5 (121.7,146.0)	135.5 (110.2,146.2)	0.005
CRP, mg/L	6.0 (2.0, 39.0)	8.5 (2.0, 41.2)	6.5 (2.0, 27.0)	12.0 (2.5, 54.0)	0.86
GFR (ml/min/1.73 m^2^)	52.5 (29.3, 70.1)	61.3 (47.7, 87.1)	67.0 (49.1, 94.4)	61.0 (49.6, 79.2)	0.002
Serum creatinine (mg/dl)	1.29 (1.03, 1.95)	1.12 (0.88, 1.46)	1.09 (0.87, 1.49)	1.12 (0.87, 1.49)	0.008
Anemia on admission	43 (55.1%)	35 (31.0%)	12 (40.0%)	9 (37.5%)	0.01
STEMI	63 (80.8%)	104 (90.4%)	30 (100.0%)	23 (95.8%)	0.014
RR systolic, mmHg	91.9 (17.0)	93.1 (21.8)	84.6 (16.0)	88.0 (30.0)	0.65
RR diastolic, mmHg	61.2 (10.6)	62.3 (14.7)	56.8 (12.7)	58.7 (28.0)	0.95
RR mean, mmHg	72.6 (11.3)	73.3 (15.2)	65.9 (14.9)	70.5 (22.9)	0.81
Mechanical ventilation	39 (50.0%)	53 (46.1%)	9 (30.0%)	13 (54.2%)	0.24
Radial access	11 (14.1%)	20 (17.4%)	3 (10.0%)	0 (0.0%)	0.14
PCI LMCA	9 (11.5%)	18 (15.7%)	3 (10.0%)	6 (25.0%)	0.35
PCI LAD	43 (55.1%)	56 (48.7%)	14 (46.7%)	8 (33.3%)	0.31
PCI LCX	15 (19.2%)	19 (16.5%)	4 (13.3%)	10 (41.7%)	0.03
PCI RCA	22 (28.2%)	28 (24.3%)	12 (40.0%)	8 (33.3%)	0.36
Multivessel PCI	20 (28.2%)	30 (29.1%)	6 (20.7%)	7 (41.2%)	0.53
Mechanical circulatory support	7 (9.0%)	10 (8.7%)	2 (6.7%)	45 (20.9%)	0.36
GPI	40 (51.3%)	48 (41.7%)	15 (50.0%)	12 (50.0%)	0.57
Bivalirudin	5 (6.4%)	16 (13.95%)	10 (33.3%)	2 (8.3%)	0.003
TIMI 0/1 after PCI	12 (15.4%)	18 (15.7%)	0 (0.0%)	2 (8.3%)	0.11
Troponin, µg/L	33.6 (8.2, 77.4)	32.6 (7.4, 82.9)	39.7 (16.6, 71.4)	30.3 (8.9, 90.1)	0.67
EF (%)	31.6 (5.4)	30.9 (5.1)	30.0 (0.1)	33.1 (8.8)	0.16
Renal replacement therapy	3 (3.8%)	5 (4.3%)	0 (0.0%)	4 (16.7%)	0.029
Cerebral hemorrhage	3 (3.8%)	1 (0.9%)	0 (0.0%)	0 (0.0%)	0.29
Stent thrombosis	0 (0.0%)	5 (4.3%)	0 (0.0%)	1 (4.2%)	0.19
Bleeding	22 (28.2%)	21 (18.3%)	4 (13.3%)	12 (50.0%)	0.003
CABG during the same hospitalization	2 (2.6%)	6 (5.2%)	3 (10.0%)	3 (12.5%)	0.20
Mortality outcome					
30-day mortality	44 (56.4%)	53 (46.1%)	12 (40.0%)	6 (25.0%)	0.045
One-year mortality	55 (70.5%)	56 (48.7%)	14 (46.7%)	9 (37.5%)	0.004

BMI, body mass index; CABG, coronary artery bypass graft; CRP, C-reactive protein; GPI, GP IIb/IIIa receptor antagonist; EF, ejection fraction; GFR, glomerular filtration rate; LAD, left anterior descending artery; LCX, circumflex artery; LMCA, left main coronary artery; MI, myocardial infarction; PCI, percutaneous intervention; RCA, right coronary artery; RR, blood pressure; STEMI, ST-elevation myocardial infarction; TIMI, thrombolysis in myocardial infarction.

Data are expressed as mean ± SD, a number (percentage), or the median (interquartile range).

### In-hospital bleeding

**Figure 1:**
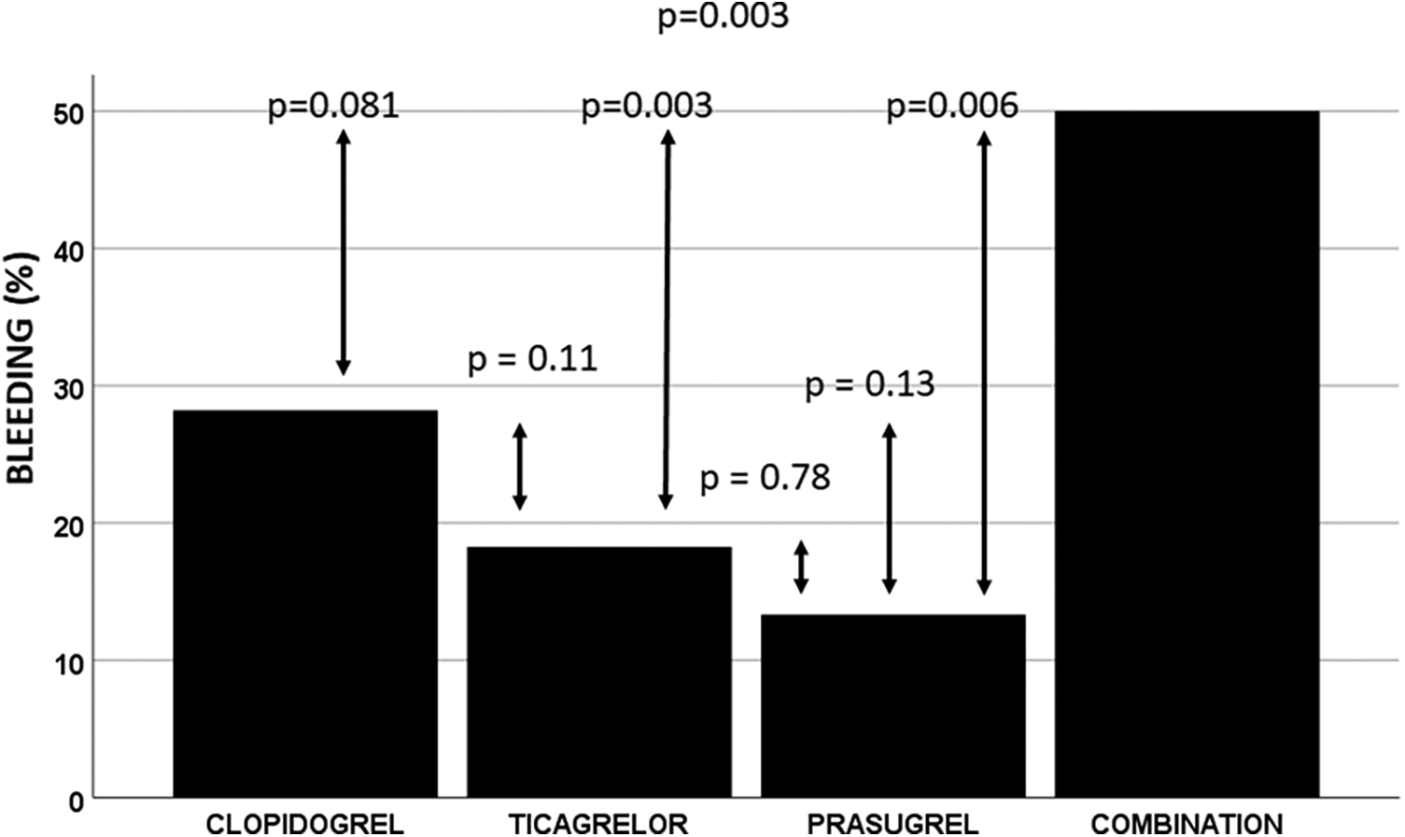
Observed in-hospital bleeding rate. Combination, P2Y12 combination. The in-hospital bleeding rate was highest in the P2Y12 combination group. It was similar to that in the clopidogrel group and more frequent than in the ticagrelor and prasugrel groups. A similar bleeding rate was observed in patients receiving only one P2Y12.

Bleeding occurred in 59(23.9%) patients. The highest bleeding rate was observed in patients in the P2Y12 combination group, followed by the clopidogrel, ticagrelor, and prasugrel groups [12(50%) patients, 22(28.2%), 21(18.3%), and 4(13.3%) patients experienced bleeding in the combination, clopidogrel, ticagrelor and prasugrel groups, respectively, *p* = 0.003) (Figure 1)]. Bleeding had a similar frequency in the P2Y12 combination group and the clopidogrel group (*p* = 0.081) but was more frequent than in the ticagrelor and prasugrel groups (*p* = 0.002 and *p* = 0.006, respectively) (Figure 1). However, when patients who received only one P2Y12 were compared, the bleeding rate was similar in the clopidogrel, ticagrelor, and prasugrel groups (*p* = 0.13). The bleeding rate was also similar when only potent P2Y12 (prasugrel, ticagrelor) were compared (*p* = 0.78). Bleeding was associated with anemia on admission (and lower hemoglobin), low GFR, age, resuscitation before PCI, mechanical ventilation, and P2Y12. In addition, stent thrombosis and renal replacement therapy during hospitalization were associated with bleeding (Supplementary Table S1).

After adjustment for confounders, P2Y12 were associated with bleeding (*p* = 0.003) (Table 2). Compared to clopidogrel, both ticagrelor, and prasugrel had a lower bleeding risk (aOR: 0.40; 95% CI: 0.18–0.92; *p* = 0.032 and aOR: 0.20; 95% CI: 0.05–0.85; *p* = 0.029, respectively), but the P2Y12 combination group had a similar bleeding risk (aOR: 2.31; 95% CI: 0.71–7.48; *p* = 0.16) (Table 2). Mechanical ventilation, age, GFR, and anemia on admission were also associated with bleeding. In contrast, GP IIb/IIIa receptor antagonists, bivalirudin, and oral anticoagulants were not associated with bleeding (Table 2). Ticagrelor and prasugrel patients had more than an 80% and 90% lower risk of bleeding compared to the P2Y12 combination group when the P2Y12 combination group was used as a reference (aOR: 0.17; 95% CI: 0.06–0.55; *p* = 0.003 and aOR: 0.09; 95% CI: 0.02–0.44; *p* = 0.003; respectively).

**Table 2:** Association with bleeding.

Variable	Multivariable model	*p*
	aOR (95% CI)	
Mechanical ventilation	2.53 (1.04–6.16)	0.042
Resuscitation prior to PCI	1.01 (0.42–2.44)	0.97
Male sex	0.90 (0.43–1.90)	0.78
Radial access	1.28 (0.47–3.51)	0.63
P2Y12^a^		0.003
Ticagrelor	0.40 (0.18–0.92)	0.032
Prasugrel	0.20 (0.05–0.85)	0.029
P2Y12 combination	2.31 (0.71–7.48)	0.16
GPI	1.002 (0.481–2.089)	0.99
Bivalirudin	2.34 (0.83–6.60)	0.11
Oral AC therapy	0.53 (0.14–2.09)	0.37
Anemia on admission	3.44 (1.55–7.67)	0.002
Age, years	0.96 (0.93–0.99)	0.015
GFR (ml/min/1.73 m^2^)	0.98 (0.96–0.99)	0.003
Renal replacement therapy	1.21 (0.30–4.94)	0.80

AC, anticoagulant therapy; aOR, adjusted odd ratio; CI, confidence interval; GFR, glomerular filtration rate; GPI, GP IIb/IIIa receptor antagonist; P2Y12, P2Y12 receptor antagonist.

^a^ Clopidogrel group as reference.

### Mortality

**Figure 2:**
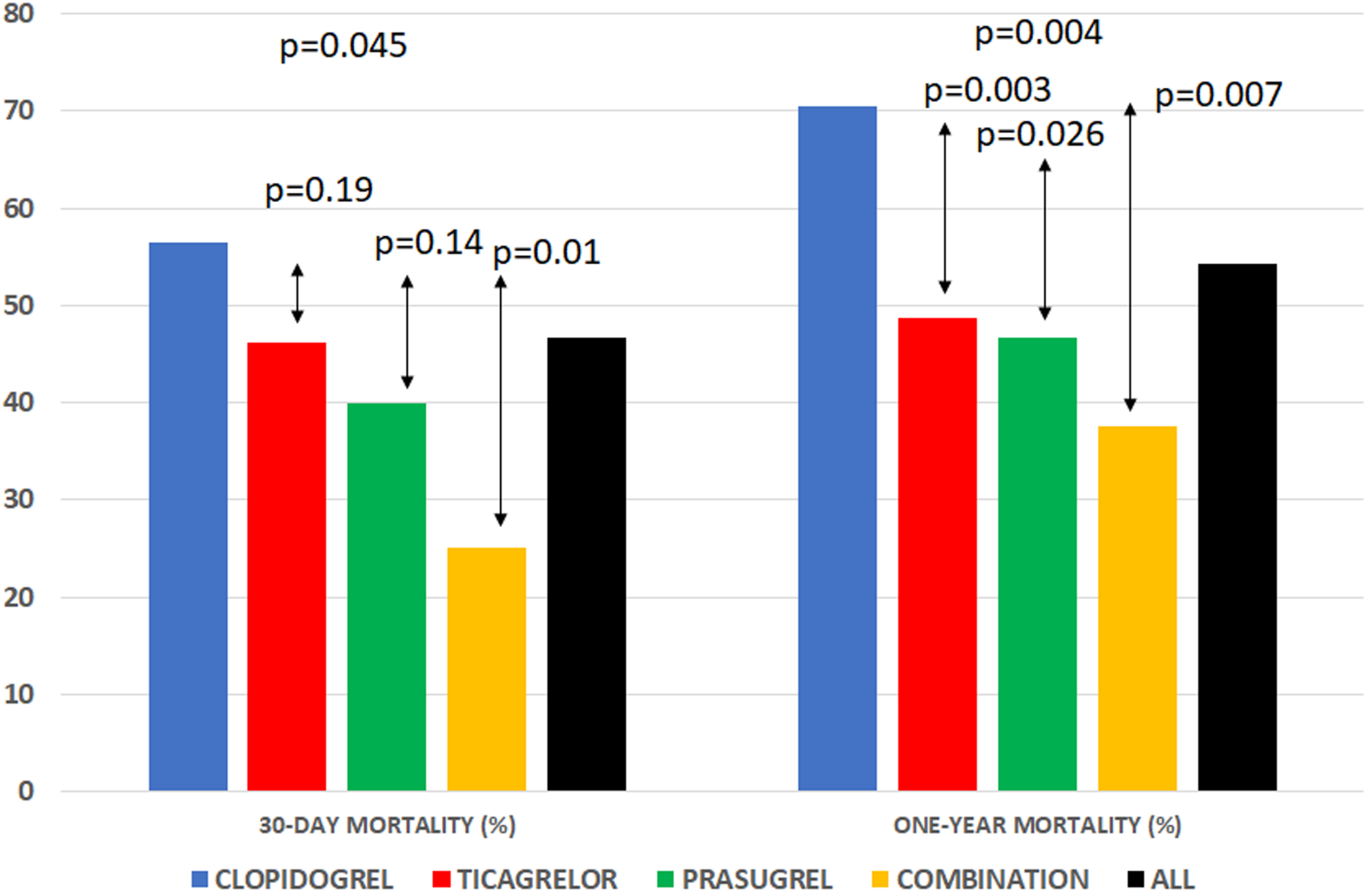
Unadjusted 30-day all-cause mortality was highest in the clopidogrel group, followed by the ticagrelor, prasugrel, and P2Y12 combination groups. Patients who received only one P2Y12 had similar mortality, and only patients in the P2Y12 combination group had lower mortality than the clopidogrel group. After one year, the highest all-cause mortality rate was observed in patients receiving clopidogrel. The groups with ticagrelor, prasugrel, and P2Y12 combinations had a lower one-year mortality rate.

After 30 days 115(46.6%) patients had died. Unadjusted 30-day all-cause mortality were highest in the clopidogrel group, followed by the ticagrelor, prasugrel, and P2Y12 combination groups [44(56.4%) patients died in the clopidogrel group, 53(46.1%) died in the ticagrelor group, 12(40%) patients died in the prasugrel group, and 6(25%) patients died in the P2Y12 combination group within 30 days, respectively; *p* = 0.045] (Figure 2). Only patients in the P2Y12 combination group had lower observed 30-day mortality than the clopidogrel group (*p* = 0.01) (Figure 2). Patients who received only one P2Y12 had similar mortality (*p* = 0.21). The P2Y12 were not associated with 30-day mortality. Age, mechanical ventilation, resuscitation prior to PCI, GFR, anemia, blood pressure on admission, PCI of right or circumflex artery, TIMI 0/1 after PCI were associated with 30-day mortality (Supplementary Table S1).

**Figure 3:**
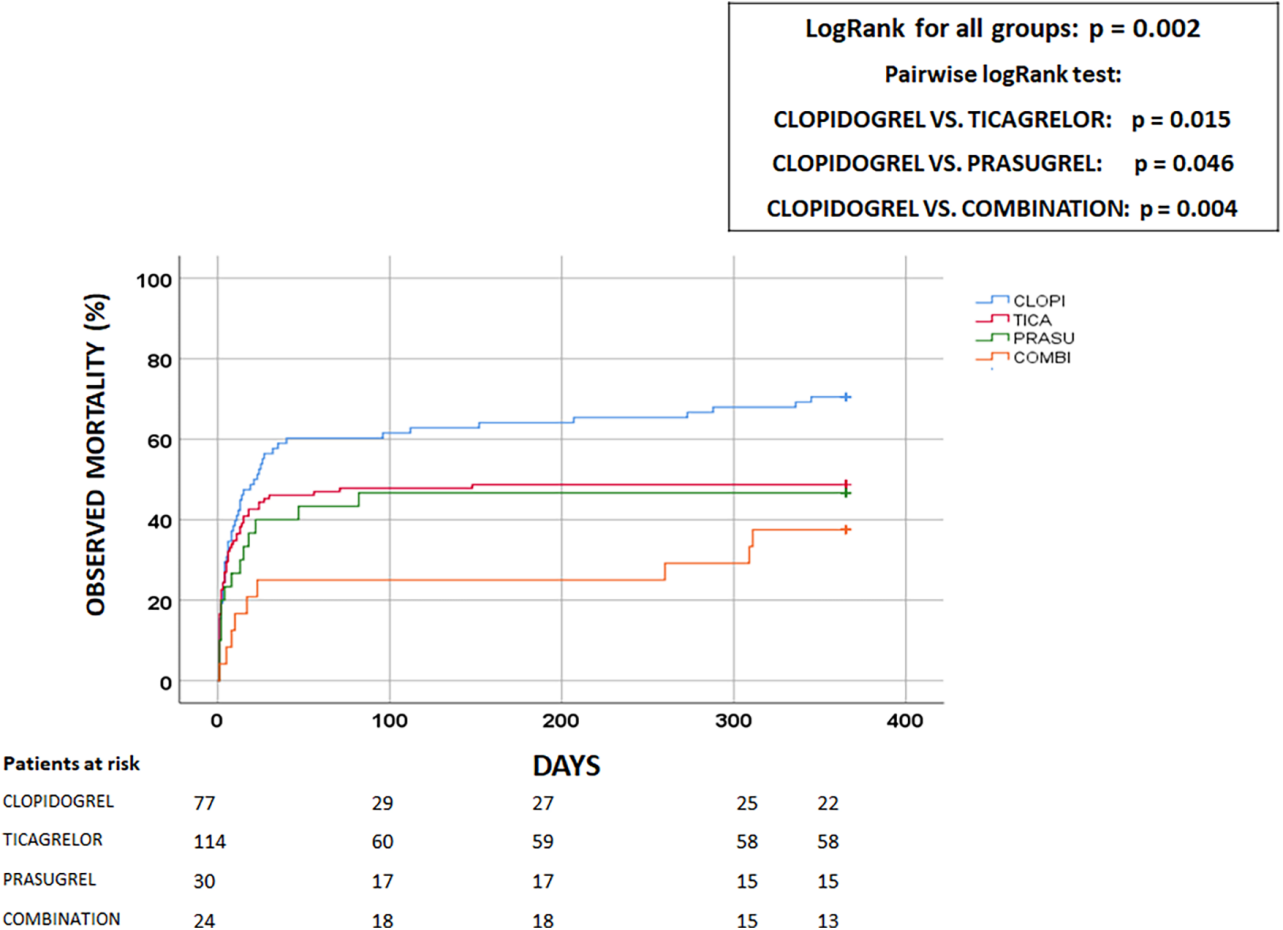
Estimated all-cause one-year mortality. Clopi, clopidogrel; Prasu, prasugrel; Tica, ticagrelor; Combination, P2Y12 receptor antagonist combination. The unadjusted one-year all-cause mortality was lowest in the P2Y12 combination group, followed by the prasugrel, ticagrelor, and clopidogrel groups. The pairwise logrank comparison showed significantly higher estimated mortality in the clopidogrel group compared with the other groups.

After one year, 134(54.3%) patients had died. The highest all-cause mortality rate was observed in patients on clopidogrel [55(70.5%)], followed by patients on ticagrelor, prasugrel, and a P2Y12 combination (56(48.7%), 14(46.7%) and 9(37.5%) patients died, respectively; *p* = 0.004) (Figure 2). The cumulative incidence rates of unadjusted one-year mortality by the Kaplan-Meier method showed a significant difference between groups (logrank = 0.002) (Figure 3). The pairwise logrank comparison showed a significantly higher estimated mortality in the clopidogrel group compared to the other groups (*p* = 0.015 compared to ticagrelor, *p* = 0.046 compared to prasugrel, and *p* = 0.004 compared to the P2Y12 combination, respectively) (Figure 3). In addition, this pairwise comparison showed a similar estimated mortality in the ticagrelor, prasugrel, and P2Y12 combination groups (*p* = 0.72 for ticagrelor vs. prasugrel, *p* = 0.20 for ticagrelor vs. P2Y12 combination, and *p* = 0.37 for prasugrel vs. P2Y12 combination). Age, mechanical ventilation, resuscitation prior to PCI, GFR, anemia, blood pressure on admission, PCI of right coronary artery, TIMI 0/1 after PCI, hyperlipidemia, hypertension, and P2Y12 were associated with one-year mortality (Supplementary Table S1).

However, after adjustment for confounding factors, the P2Y12 groups were not independently associated with either 30-day (*p* = 0.23) or one-year mortality risk (*p* = 0.36). Resuscitation prior to PCI and systolic blood pressure were independently associated with 30-day mortality risk, while only systolic blood pressure was associated with one-year mortality risk (Table 3).

**Table 3:** Association with 30-day and one-year mortality.

Variable	30-day mortality^a^		One-year mortality^a^	
	aOR (95% CI)	*p*	aHR (95%CI)	*p*
Age	1.02 0.97–1.08)	0.40	1.01 (0.99–1.04)	0.26
Male sex	0.69 (0.18–2.64)	0.59	0.99 (0.54–1.84)	0.98
Diabetes mellitus	0.55 (0.11–2.80)	0.47	1.21 (0.58–2.50)	0.61
Hypertension	0.52 (0.11–2.50)	0.41	0.64 (0.32–1.32)	0.57
Hyperlipidemia	5.84 (0.55–61.57)	0.14	1.30 (0.52–3.25)	0.57
GFR	0.98 (0.96–1.01)	0.18	0.99 (0.98–1.01)	0.32
Anemia on admission	4.30 (0.90–20.65)	0.068	1.07 (0.56–2.02)	0.84
Resuscitation prior to PCI	4.45 (1.07–18.54)	0.040	1.81 (0.97–3.37)	0.063
Mechanical ventilation	0.33 (0.03–3.09)	0.33	1.01 (0.45–2.26)	0.98
RR systolic on admission	0.96 (0.0.93–0.99)	0.019	0.97 (0.96 -0.98)	<0.0001
PCI LCX	059 (0.13–2.66)	0.49	1.28 (0.63–2.61)	.0.49
PCI RCA	0.25 (0.06–1.09)	0.054	0.57 (0.29–1.15)	0.12
TIMI 0/1 after PCI	6.79 (0.93–49.54)	0.059	1.55 (0.77–3.10)	0.22
P2Y12 group^a^		0.23		0.36
Ticagrelor	4.26 (0.90–20.18)	0.068	1.42 (0.74–2.73)	0.30
Prasugrel	3.58 (0.37–34.64)	0.27	1.76 (0.61–5.10)	0.30
P2Y12 combination	0.97 (0.10–9.44)	0.98	0.66 (0.23–1.93)	0.45
Bleeding	1.51 (0.41–5.61)	0.53	1.21 (0.61–2.42)	0.58

aHR, adjusted hazard ratio; aOR, adjusted odd ratio; CI, confidence interval; GFR, glomerular filtration rate; LCX, circumflex artery; P2Y12, P2Y12 receptor antagonist; PCI, percutaneous coronary intervention; RCA, right coronary artery; RR, blood pressure; TIMI, thrombolysis in myocardial infarction.

^a^ Clopidogrel as reference.

## Discussion

There are no data on the potential effects of the combination of more than one P2Y12 in patients with cardiogenic shock due to MI compared with a single P2Y12 (clopidogrel, ticagrelor, or prasugrel) on bleeding and outcome. We retrospectively analyzed 247 patients with MI and CS who received either clopidogrel, ticagrelor, prasugrel, or a combination of P2Y12. The main results of our analysis are as follows:

1. There was no significant difference in the multivariable-adjusted all-cause 30-day and one-year mortality risk between patients receiving clopidogrel, ticagrelor, prasugrel, and a combination of P2Y12.
2. Patients receiving a combination of P2Y12 had a significantly higher unadjusted bleeding rate than patients receiving ticagrelor or prasugrel but a similar rate to that of patients receiving clopidogrel.
3. Ticagrelor and prasugrel patients had a lower bleeding risk after adjustment for confounders than patients receiving a combination of P2Y12 or clopidogrel.

Our results suggest that the choice of P2Y12 in patients with MI and CS has no significant effect on mortality but may influence the risk of bleeding.

As previously observed, almost half of the patients were mechanically ventilated on admission (2). The percentage of patients receiving a combination of P2Y12 in our analysis (9.7%) was comparable to the pooled analysis of two randomized controlled prospective trials (13.1%) (2). Results of previous analyzes are inconclusive and sometimes contradictory (2, 3, 9, 11, 19–21). Retrospective analyzes and pooled data showed superiority of potent P2Y12 (3, 12, 9), whereas prospective and some retrospective studies showed no difference in survival between P2Y12 (2, 11, 19–21). There were substantial differences between studies in terms of the number of patients, patient selection, end points, and variables included in the multivariable analyzes. Our previous observation also showed a different result (3). However, in our previous study, we had included a smaller number of patients, including patients who were resuscitated only (and did not suffer from CS after resuscitation), we did not have data on anemia and GFR, and the definition of bleeding was different. These differences might justify a different outcome. Our results cautiously support the previous data of Orban et al, who found better observed mortality with potent P2Y12 but no independent association between P2Y12 and mortality (2).

We confirmed the previous findings in a similar group of patients that ticagrelor is associated with a lower risk of bleeding than clopidogrel (2). In addition, we showed that prasugrel was also associated with a lower bleeding risk, and both drugs had a lower bleeding risk than the P2Y12 combination, whose bleeding risk was comparable to that of clopidogrel. There was no difference in bleeding rates between ticagrelor and prasugrel, as previously observed in patients with acute coronary syndrome without cardiogenic shock (22).

When comparing the individual groups, we found that the P2Y12 combination group received oral anticoagulants more frequently than the prasugrel and ticagrelor groups (*p* = 0.034 and *p* = 0.030, respectively), and with a similar frequency to the clopidogrel group (*p* = 0.73). However, oral anticoagulants were not associated with bleeding in the multivariable model (Table 2), which cannot explain the higher bleeding rate. TIMI flow after PCI was similar to the other groups and stent thrombosis was similar in all groups (Table 1). Patients in the P2Y12 combination group had significantly more PCI of the left circumflex artery, which was associated with a better 30-day outcome in univariable, but not in multivariable analysis (Table 3). In addition, peak troponin was similar in all groups, so infarct size was most probably not responsible for better survival. We also examined surgeries performed during the same hospitalization, and their frequencies were similar in all groups (Table 1). The Kaplan-Meier survival curve showed that the vast majority of patients died within the first 30 days (Figure 3). In addition, patients receiving a combination of P2Y12 were younger than those on clopidogrel and ticagrelor (*p* < 0.0001 and *p* = 0.038, respectively) and they received less bivalirudin than those on prasugrel (*p* = 0.048).

The clopidogrel patients were resuscitated more frequently prior to PCI compared to the prasugrel group (*p* = 0.018) and tended to be resuscitated more frequently than the ticagrelor group (*p* = 0.056). They had significantly lower hemoglobin and GFR values on admission, both known factors associated with bleeding and treatment outcome (15, 23, 24), and were more likely to receive oral AC than patients receiving ticagrelor and prasugrel. In addition, they were significantly older.

The most interesting finding of our analysis was that patients with a P2Y12 combination bled significantly more often than patients with ticagrelor and prasugrel, but similarly to patients with clopidogrel. Several reasons could account for this bleeding tendency in the P2Y12 combination group. Patients with MI and CS are more prone to bleeding than other MI patients, mainly because of more aggressive treatment such as resuscitation, mechanical circulatory support, and frequent punctures of arteries and central veins (2). In patients with CS, drug absorption, biotransformation, bioavailability, and excretion are slower than in other patients with MI (3, 5–7, 10). Therefore, it can be assumed that the active ingredient of the previously administered P2Y12 remains active in the body even if the P2Y12 is changed according to the recommendations, which could explain the very high bleeding rate in the P2Y12 combination group. Unfortunately, we lack data on platelet reactivity.

Although the bleeding rate was higher in our analysis, the mortality rate was comparable to previous observations (2). As already mentioned, P2Y12 were not associated with outcome after adjustment for confounding factors. Only systolic blood pressure at admission was associated with 30-day and 1-year mortality (Table 3). In addition, resuscitation prior to PCI was associated with 30-day mortality. This may suggest that bleeding itself is less important in this particular patient group compared to other variables than in other patients with MI. We can only speculate that in this particular group of patients, so many vital systems are damaged that anemia and bleeding are less important to the outcome than resuscitation and systolic blood pressure on admission, which might better reflect the patient's situation.

Our finding supports previous observations in similar patients in whom the higher bleeding rate did not result in a worse outcome (2). Moreover, these patients had the lowest crude 30-day and one-year mortality rates, which were significantly lower than in the clopidogrel group and similar to those in the ticagrelor and prasugrel groups (Figures 2, 3). This phenomenon remains unexplained (2).

A unique and novel finding of the present study is that the combination of P2Y12 is associated with a significantly higher risk of bleeding in the acute phase of MI with CS, which surprisingly does not lead to a worse outcome. Our data may have some clinical implications. Based on our results, it seems reasonable to wait longer than recommended before switching between P2Y12 in patients with CS. Further research is needed to determine whether platelet reactivity should be measured before switching to P2Y12 to avoid the high risk of bleeding in these patients.

Our results point to the complex relationship between treatment, bleeding, and mortality in MI patients with CS and suggest that it may be difficult to account for all possible confounding factors in these patients. The lack of association between bleeding and outcome could be due to a still unidentified specific factor related to cardiogenic shock or to unknown confounding factors that were not considered in our analysis.

Our results suggest that the combination of P2Y12 increases the risk of bleeding by 80% and 90%, respectively, compared with ticagrelor and prasugrel, but this does not translate into a worse outcome in patients with MI and CS. The possible pathomechanisms to explain the “benign” bleeding in these patients are unclear, and they were not investigated in this study, but we did propose several hypotheses. Further research is needed to determine the still unclear pathophysiological mechanisms, which are probably multifactorial.

## Limitations

Our analysis has relevant limitations. It was a retrospective study at a single center and therefore provides only associative, not causal, evidence. The major limitation is the small sample size, especially when subgroups were analyzed. The number of patients might have influenced the outcome in terms of mortality. A large sample size is usually required to detect a significant difference in mortality. The population was enrolled over a long period of time, and many differences in treatment may contribute to the changes in mortality over time. However, when we included the year of admission in the multivariable analysis, it was not associated with either bleeding (*p* = 0.06) or mortality (*p* = 0.33), so it is highly unlikely that these different practices over the years had a significant impact on the outcome. Current practices were not fully accounted for in this study (nor in a previous observation), which is certainly a limitation of the study. There were fairly broad CIs in the multivariate analysis, which detracts from the power of our analysis. There were no exclusion criteria related to concomitant diseases or clinical presentation, so this population represents a real experience of high-risk patients requiring PCI.

## Conclusions

Our study results suggest that the choice of P2Y12 was not associated with treatment outcome. The combination of P2Y12 increased bleeding risk compared with ticagrelor and prasugrel and was comparable to clopidogrel in patients with MI and CS. However, these higher bleeding rates did not result in worse treatment outcomes. Our results point to the complex relationship between treatment, bleeding, and mortality in MI patients with CS and suggest that it may be difficult to account for all possible confounding factors in these patients.

## Data Availability

The raw data supporting the conclusions of this article will be made available by the authors, without undue reservation.
