# Supplementary material for: Clopidogrel, ticagrelor, prasugrel or an alternation of two P2Y12 in patients with acute myocardial infarction with cardiogenic shock
Source: Front Cardiovasc Med. 2024 Jan 3;10:1266127. doi: 10.3389/fcvm.2023.1266127 (PMC10792002; doi:10.3389/fcvm.2023.1266127)
Supplement: Supplementary file 1 [file Table1.docx]

Supplementary Table 1

Univariable associations with in-hospital bleeding, 30-day and one-year mortality

| VARIABLE | UNIVARIABLE ASSOCIATION | | | | | |
| --- | --- | --- | --- | --- | --- | --- |
|  | BLEEDING | | 30-DAY MORTALITY | | ONE-YEAR MOTALITY |  |
|  | Or (95% CI) | p | Or (95% CI) | p | Or (95% CI) | p |
| Age, years | 0.96 (0.94-0.99) | 0.004 | 1.04 (1.02-1.07) | <0.0001 | 1.04 (1.01-1.06) | 0.001 |
| Male sex | 0.85 (0.45-1.60) | 0.61 | 0.92 (0.54-1.56) | 0.75 | 1.04 (0.61-1.77) | 0.89 |
| Diabetes mellitus | 0.98 (0.48-1.99) | 0.96 | 0.94 (0.52-1.72) | 0.85 | 0.92 (0.51-1.68) | 0.80 |
| Hypertension | 1.06 (0.58-1.92) | 0.86 | 0.59 (0.35-0.99) | 0.057 | 0.57 (0.34-0.96) | 0.034 |
| Hyperlipidemia | 1.07 (0.51-2.29) | 0.85 | 0.60 (0.31-1.18) | 0.14 | 0.46 (0.24-0.90) | 0.024 |
| Previous stroke | 1.64 (0.47-5.64) | 0.43 | 1.64 (0.51-5.34) | 0.41 | 1.19 (0.37-3.86 | 0.77 |
| Chronic kidney disease | 1.62 (0.40-6.71) | 0.50 | 2.37 (0.58-9.69) | 0.23 | 3.06 (0.62-15.03) | 0.17 |
| Previous MI | 0.78 (0.21-2.88) | 0.72 | 3.38 (0.98-10.94) | 0.051 | 5.96 (1.1.32-27.01) | 0.021 |
| Previous PCI/CABG | 0.62 (0.13-2.93) | 0.55 | 3.65 (0.96-13.83) | 0.057 | 4.48 (0.96-20.87) | 0.056 |
| BMI, kg/m2 | 0.99 (0.92-1.07) | 0.83 | 0.96 (0.90-1.02) | 0.22 | 0.97 (0.90-1.03) | 0.29 |
| Hemoglobin, g/L | 1.02 (1.01-1.04 | 0.022 | 0.98 (0.97-0.99) | 0.006 | 0.98 (0.97-0.99) | 0.003 |
| Anemia on admission | 2.70 (1.39-5.26) | 0.039 | 2,70 (1.39-5.26) | 0.03 | 1.82 (1.08-3.06) | 0.024 |
| Serum creatinine (mg/dl) | 1.09 (0.85-1.40) | 0.48 | 1.67 (1.16-2.39) | 0.005 | 1.56 (1.08-2.23) | 0.017 |
| GFR (ml/min/1.73m^2^) | 0.98 (0.97-0.99) | 0.014 | 0.98 (0.97-0.99) | <0.0001 | 0.98 (0.97-0.99) | <0.0001 |
| Resuscitation before PCI | 2.19 (1.21-3.96) | 0.01 | 1.77 (1.06-2.96) | 0.028 | 1.93 (1.15-3.25) | 0.013 |
| Mechanical ventilation | 2.94 (1.59-542) | 0.001 | 1.80 (1.08-2.99) | 0.023 | 2.10 (1.26-3.51) | 0.005 |
| STEMI | 1.92 (0.63-5.79) | 0.25 | 0.67 (0.30-1.49) | 0.32 | 0.56 (0.24-1.29) | 0.17 |
| Mechanical cIrculatory.support | 1.35 (0.53-.44) | 0.52 | 1.40 (0.60-3.26) | 0.43 | 0.99 (0.43-2.32) | 0.99 |
| Systolic blood pressure on admission, mmHg | 1.02 (0.99-1.04) | 0.16 | 0.96 (0.94-0.99) | 0.006 | 0.96 (0.94-0.99) | 0.002 |
| Diastolic blood pressure on admission, mmHg | 1.004 (0.967-1.041) | 0.85 | 0.97 (0.93-1.01) | 0.085 | 0.97 (0.94-1.00) | 0.05 |
| Mean blood pressure on admission, mmHg | 1.01 (0.97-1.05) | 0.57 | 0.96 (0.93-0.99) | 0.037 | 0.97 (0.93-1.01) | 0.096 |
| Radial access | 0.98 (0.42-2.29) | 096 | 0.77 (0.37-1.62) | 0.50 | 0.72 (0.35-1.48) | 0.37 |
| PCI LMCA | 1.27 (0.57-2.82) | 0.55 | 1.17 (0.58-2.38) | 0.65 | 0.93 (0.47-1.89) | 0.85 |
| PCI LAD | 1.01(0.56-1.81) | 0.97 | 1.45 (0.88-2.39= | 0.15 | 1.52 (0.92-2.51) | 0.10 |
| PCI LCX | 1.08 (0.53-2.24) | 0.84 | 0.50 (0.26-0.98) | 0.043 | 0.59 (0.31-1.12) | 0.11 |
| RCA | 0.82 (0.42-1.60) | 0.57 | 0.45 (0.25-0.81) | 0.007 | 0.57 (0.33-0.99) | 0.049 |
| Multivessel PCI | 0.67 (0.32-1.37) | 0.27 | 0.93 (0.52-1.68) | 0.81 | 0.97 (0.54-1.74) | 0.91 |
| GPI | 0.87 (0.49-1.58) | 0.66 | 0.79 (0.48-1.31) | 0.36 | 1.11 (0.67-1.84) | 0.68 |
| Bivalirudin | 1.46 (0.65-3.28) | 0.35 | 0.53 (0.24-1.14) | 0.11 | 0.66 (0.32-1.39) | 0.28 |
| TIMI 0/1 after PCI | 1.13 (0.80-1.60) | 0.47 | 4.09 (1.76-9.51) | 0.001 | 3.47 (1.44-8.37) | 0.006 |
| Troponin (peak), µg/L | 1.005 (0.999-1.010) | 0.083 | 1.002 (0.997-1.007) | 0.36 | 1.002(0.997-1.007) | 0.47 |
| Renal replacement therapy | 3.43(1.06-11.09) | 0.039 | 1.65 (051-5.34) | 0.41 | 1.19 (0.37-3.86) | 0.77 |
| Stent thrombosis | 6.67 (1.21-37.92) | 0.03 | 2.34 (0.42-13.03) | 0.33 | 4.34 (0.50-37.71) | 0.18 |
| P2Y12* |  | 0.006 |  | 0.053 |  | 0.005 |
| Ticagrelor | 0.57 (0.29-1.13) | 0.10 | 0.66 (0.37-1.18) | 0.16 | 0.39 (0.22-0.72 | 0.003 |
| Prasugrel | 0.39 (0.12-1.25) | 0.11 | 0.51 (0.22-1.21) | 0.13 | 0.37 (0.15-0.87) | 0.023 |
| Combination | 2.54 (0.99-6.52) | 0.051 | 0.26 (0.09-0.72) | 0.01 | 0.25 (0.10-0.65) | 0.005 |
| Bleeding |  |  | 1.50 (0.83-2.72) | 0.18 | 1.09 (0.61-1.97) | 0.76 |
| EF (%) | 1.02 (0.96-1.07) | 0.55 | 0.99 (0.95-1.04) | 0.74 | 1.002 (0.956-1.052) | 0.92 |
| Oral AC therapy | 0.96 (0.44-2.08) | 0.91 | 1.16 (0.44-3.03) | 0.76 | 1.75 (0.64-4.83) | 0.28 |
| CABG in the same hospitalization | 0.86 (0.23-3.20) | 0.82 | 0.62 (0.20-1.91) | 0.41 | 0.61 (0.21-1.83) | 0.38 |

*Clopidogrel as reference

AC therapy = anticoagulant therapy; BMI = body mass index; CABG = coronary artery by-pass graft; EF = ejection fraction; GPI = GP IIb/IIIa receptor antagonist; GFR = glomerular filtration rate; LAD = left anterior descending artery; LCX = circumflex artery; LMCA = left main coronary artery; MI = myocardial infarction; P2Y12 = P2Y12 receptor antagonist; PCI = percutaneous intervention; RCA = right coronary artery; STEMI = ST-elevation myocardial infarction; TIMI = Thrombolysis In Myocardial Infarction.
